# ZnO Nanorod/Ag Nanoparticle—Functionalized Paper Substrate for Sensitive SERS Detection of Environmental Contaminants

**DOI:** 10.1021/acsanm.5c01103

**Published:** 2025-05-14

**Authors:** Maíza Ozório, Ana Pimentel, Maria Morais, Mariana Cortinhal, Rafael Jesus Gonçalves Rubira, Tatiana Aparecida Oliveira, Jonas Deuermeier, Hugo Águas, Luis M. N. Pereira, Carlos José Leopoldo Constantino, Rodrigo Martins

**Affiliations:** † Department of Physics, School of Science and Technology (FCT), São Paulo State University − UNESP, Presidente Prudente, São Paulo 19060-900, Brazil; ‡ Department of Materials Science, NOVA School of Science and Technology and CEMOP/UNINOVA, i3N/CENIMAT, Campus de Caparica, Caparica 2829-516, Portugal; § Physics Department, Institute of Geosciences and Exact Sciences (IGCE), Sao Paulo State University − UNESP, Rio Claro, São Paulo 13506-900, Brazil

**Keywords:** paper substrate, SERS, ZnO NRs, pesticides, sensors, environmental contaminants

## Abstract

In this work, we functionalize Whatman no 1 paper (WP) substrates with zinc oxide nanorods (ZnO NRs) decorated with silver nanoparticles (AgNPs) for detecting environmental contaminants. ZnO NRs were grown by hydrothermal synthesis assisted by microwave irradiation on WP substrate and subsequently decorated with AgNPs using the dewetting deposition method. WP substrates functionalized with ZnO NRs/AgNPs were used as a SERS platform for detecting rhodamine 6G (R6G) dye and the thiabendazole (TBZ) pesticide, widely used in modern agriculture for fungal disease protection in fruits and vegetables. Additionally, a mixture of TBZ and carbendazim (CBZ) was used to evaluate the selectivity of the substrates. WP substrates with ZnO NRs/AgNPs demonstrated superior SERS performance than those with only AgNPs. The set of substrates analyzed also demonstrated high reproducibility and stability for detecting environmental contaminants. The enhanced detection with ZnO NRs is attributed to their large surface area, facilitating target molecule adsorption and increasing interactions with incident light, thus improving sensitivity. For R6G, the enhancement factors (EF) obtained using AgNPs and ZnO NRs/AgNPs were 9.3 × 10^3^ and 2.1 × 10^5^, respectively, while the limits of detection (LOD) were approximately 4.8 × 10^–8^ and 4.3 × 10^–9^ mol/L. For the pesticide TBZ, the EF values using AgNPs and ZnO NRs/AgNPs were 2.0 × 10^5^ and 9.8 × 10^6^, with corresponding LODs of 4.0 × 10^–8^ and 5.0 × 10^–10^ mol/L, respectively. ZnO NRs/AgNPs substrates also demonstrated long-term stability and selectivity for detecting pesticide mixtures, reinforcing the potential of paper-based SERS platforms for environmental monitoring. The paper substrate offers several advantages, including ease of handling, flexibility, sustainability, and low cost. Moreover, its ease of chemical functionalization enables the integration of various nanomaterials, thereby expanding its potential for a wide range of applications, including environmental and biological monitoring, food safety, and beyond.

## Graphical abstract

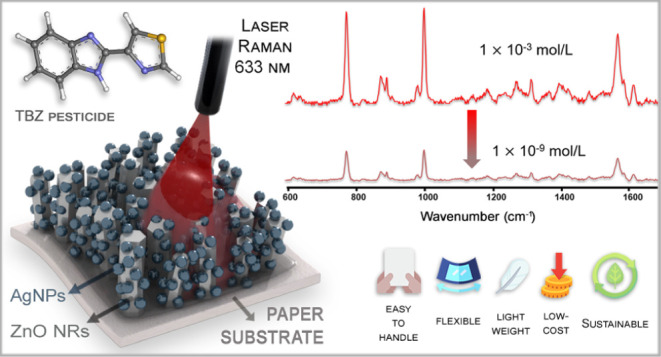

## 1 Introduction

Over the past years, paper has emerged as a material of significant interest to researchers. Its appeal lies in its flexibility, physical-chemical properties, nontoxicity, cost-effectiveness, easy functionalization, recyclability and environmental friendliness.^1–3^ The applications of paper substrates span from flexible electronic devices^4,5^ to analyte detection via Surface-Enhanced Raman Scattering (SERS).^6–8^ Notably, paper-based SERS substrates have been developed and have garnered significant attention from researchers in this field.^9,10^ Researchers have found extensive use in the ultrasensitive detection of organic molecules, particularly environmental contaminants like pesticides,^11^ drugs,^12^ and antibiotics,^13^ underscoring the advantages of paper in this research area.

The SERS effect enables the detection of extremely low concentrations of analytes,^14–16^ offering sensitivity and selectivity to the analyses. The technique is considered a highly promising analytical tool in various fields, including environmental monitoring, food safety, surface characterization, chemical detection, and medical diagnostics, due to its molecular specificity and rapid response.^17^ Sensitivity stems from the factors amplifying the Raman signal, which can reach 10^10^ under specific conditions, such as favorable adsorption mechanisms of the target molecule on the surface of metallic nanoparticles and in interstitial regions between nanoparticles (“hot spots”).^18^ In this way, paper-based SERS can significantly improve the Raman signal (Enhancement Factor (EF) ≈ 10^5^–10^7^).^19,20^ However, to ensure effective detection, the functionalization of these substrates is crucial.^21^ This adaptation involves treating the paper surface, followed by the deposition of metallic nanoparticles (MNPs) or immersion of the paper in a solution containing such MNPs. Other techniques include immersion of the paper in the analyte of interest with metallic nanoparticles, among others.

Plasmonic nanohybrids have emerged as a powerful strategy to boost the performance of SERS-based sensing platforms.^22–24^ These nanostructures typically combine metallic nanoparticles such as silver (Ag) or gold (Au) with other functional materials, including semiconductors such as zinc oxide (ZnO) or titanium dioxide (TiO_2_).^25–27^ The interaction between the components of the nanohybrid promotes the formation of intense electromagnetic “hot spots”, enhances analyte adsorption, and improves the structural stability and reproducibility of the substrate. Yi et al.^28^ reported a two-step method for preparing large-scale, vertically aligned zinc oxide nanorods (ZnO NRs) decorated with silver nanoparticles (AgNPs), resulting in highly sensitive and uniform SERS substrates. The nanostructures were produced using the magnetron sputtering method on glass substrates and applied to detect 4-aminothiophenol (4-ATP).

Pham et al.^29^ reported the hydrothermal growth of two-dimensional 2D zinc oxide nanoplates (ZnO NPls) and the uniform deposition of AgNPs on the surface of ZnO NPls through the silver nitrate reduction procedure with sodium borohydride to create a semiconductor/AgNPs hybrid structure. The SERS effect was investigated using methylene blue (MB) as the target molecule. The maximum EF value for 10^–4^ mol/L of MB reached 6.2 × 10^6^ for the peak at 1436 cm^–1^ and the limit of detection (LOD) was 10^–9^ mol/L. Xu et al.^30^ reported a TiO_2_/Ag-based SERS bioprobe with ultrahigh sensitivity, good specificity, low toxicity, and high accuracy in detecting circulating tumor cells (CTCs). The significant improvement in the SERS signal was due to the contributions of three main effects: (i) enhancement of the local electromagnetic field provided by MNPs, (ii) increase in the surface area provided by semiconductor nanostructures, and (iii) chemical enhancement supported by semiconductors caused by charge transfer between the metal and the semiconductor.^31,32^ Although high SERS efficiency can be achieved in many cases, most of the substrates reported in the literature are still silicon-based.

In recent years, several authors have reported the functionalization of paper substrates with semiconductor nanostructures for use as SERS platforms.^33,34^ Compared to conventional silicon-based substrates, the paper offers several compelling advantages for SERS applications. Its porous and fibrous structure provides a large surface area, which facilitates greater adsorption of molecules, and the ease of chemical functionalization enables the integration of various nanomaterials, such as metallic nanoparticles and semiconductor nanostructures, allowing for the customized design of efficient SERS substrates. Moreover, paper is lightweight, flexible, low-cost, and biodegradable, making it an attractive platform for the development of portable and disposable sensing devices. These features make paper-based SERS platforms particularly well-suited for on-site and real-time analysis.

ZnO is one of the most used semiconductors for paper-based SERS functionalization^35,36^ due to the unique combination of high surface area tunability, chemical stability, biocompatibility, and ease of synthesis into diverse morphologies. These appealing features make this metal oxide ideal for a variety of analytical applications.^37,38^ In summary, paper substrates functionalized with ZnO are attractive due to their cost-effectiveness, versatility, functionalization capacity, and environmental and biological compatibility. Therefore, these substrates are increasingly explored for developing sensitive and affordable detection devices, highlighting their potential for future research. In this work, we demonstrated the microwave-assisted hydrothermal growth of ZnO NRs on a Whatman paper (WP) substrate and their subsequent decoration with AgNPs by thermal evaporation assisted by an electron beam (dewetting method). WP functionalized with ZnO NRs/AgNPs were used as SERS substrates to detect the pesticide thiabendazole (TBZ). In addition to detection, the substrate showed long-term stability and selectivity between the pesticides TBZ and carbendazim (CBZ). Selectivity is an essential characteristic for the effectiveness and precision of sensors, positively impacting the application of environmental monitoring. TBZ and CBZ are two of the most widely used benzimidazole fungicides in preventing fungal diseases in fruits and vegetables for preharvest and postharvest protection in agriculture.^39^

## 2 Experimental Section

### 2.1 Materials

Whatman chromatography (WP) grade 1 paper (Whatman International Ltd., Florham Park, NJ, USA) substrates were cut to 20 × 25 mm dimensions. Zinc oxide nanorods (ZnO NRs) were synthesized using an aqueous solution containing 25 mM zinc nitrate hexahydrate (Zn­(NO_3_)_2_·6H_2_O, 98%) and 25 mM hexamethylenetetramine (C_6_H_12_N_4_, 99%). Tetraethylrhodamine hydrochloride (Rhodamine 6G—R6G), thiabendazole (TBZ—C_10_H_7_N_3_S, 99%), and carbendazim (CBZ—C_9_H_9_N_3_O_2_, 98%) were selected as target analytes for the investigation. Ultrapure water (ρ = 18.2 MΩ), obtained from the Milli-Q water system, has been used in all experiments. All materials were purchased from Sigma–Aldrich.

### 2.2 Synthesis of ZnO NRs

ZnO NRs were synthesized following the procedure described by Pimentel et al.^40^ Initially, a ZnO seed layer was deposited onto WP substrates via radio frequency (RF) sputtering in an AJA ATC-1300F system without intentional substrate heating. A high-purity ceramic oxide target with a purity of 99.99%, supplied by Alineason Materials Technology GmbH, was utilized for this deposition process. Before the ZnO deposition, the chamber was evacuated to a base pressure of 10^–6^ Torr to ensure cleanliness. The deposition parameters included a power density of 4.2 W cm^–2^, a 2.3 mTorr deposition pressure, an argon and oxygen atmosphere, and a fixed target-to-substrate distance of 18 cm. The deposition duration was 122 min, forming a 200 nm thick ZnO layer on the WP substrates. Following the deposition, the WP substrates coated with the ZnO seed layer underwent ultraviolet (UV) treatment for 5 min using a UV/Ozone system from Novascan (Bonne, MO, USA), equipped with UV lamps emitting 185 and 254 nm wavelengths. The distance between the WP substrate and UV lamps was maintained at 10 cm during the treatment. The UV treatment aims to improve the vertical alignment of the ZnO nanorods. Essentially, this process promotes the decomposition of organic species and the formation of hydroxyl radicals on the ZnO surface, enhancing the film’s wettability and enabling the hydrothermal growth of a homogeneous ZnO nanorod layer. Additionally, the degradation of organic species leads to the formation of shallow donor levels, which, together with changes in oxygen vacancies caused by oxygen radicals generated in the UV-treated ZnO structures, modify the carrier concentration in the film.^41^ As a result, the combined effects of UV treatment can lead to the growth of longer ZnO nanorods under hydrothermal conditions. Subsequently, ZnO nanorod arrays were synthesized via hydrothermal synthesis under microwave irradiation using a Discover SP microwave system from CEM (Matthews, NC, USA). The WP substrates were positioned at an angle against the Pyrex vessel, with the seed layer facing downward. The vessel was filled with an aqueous solution containing 25 mM zinc nitrate hexahydrate and 25 mM hexamethylenetetramine. The synthesis was conducted with a microwave power input of 50 W and a constant temperature of 100 °C for 10 min. After each synthesis cycle, the paper substrates were cleaned with deionized water and dried on a heated plate at 60 °C. A schematic diagram illustrating the production process of ZnO NRs arrays on WP substrates is shown in Figure 1a.

**Figure 1:**
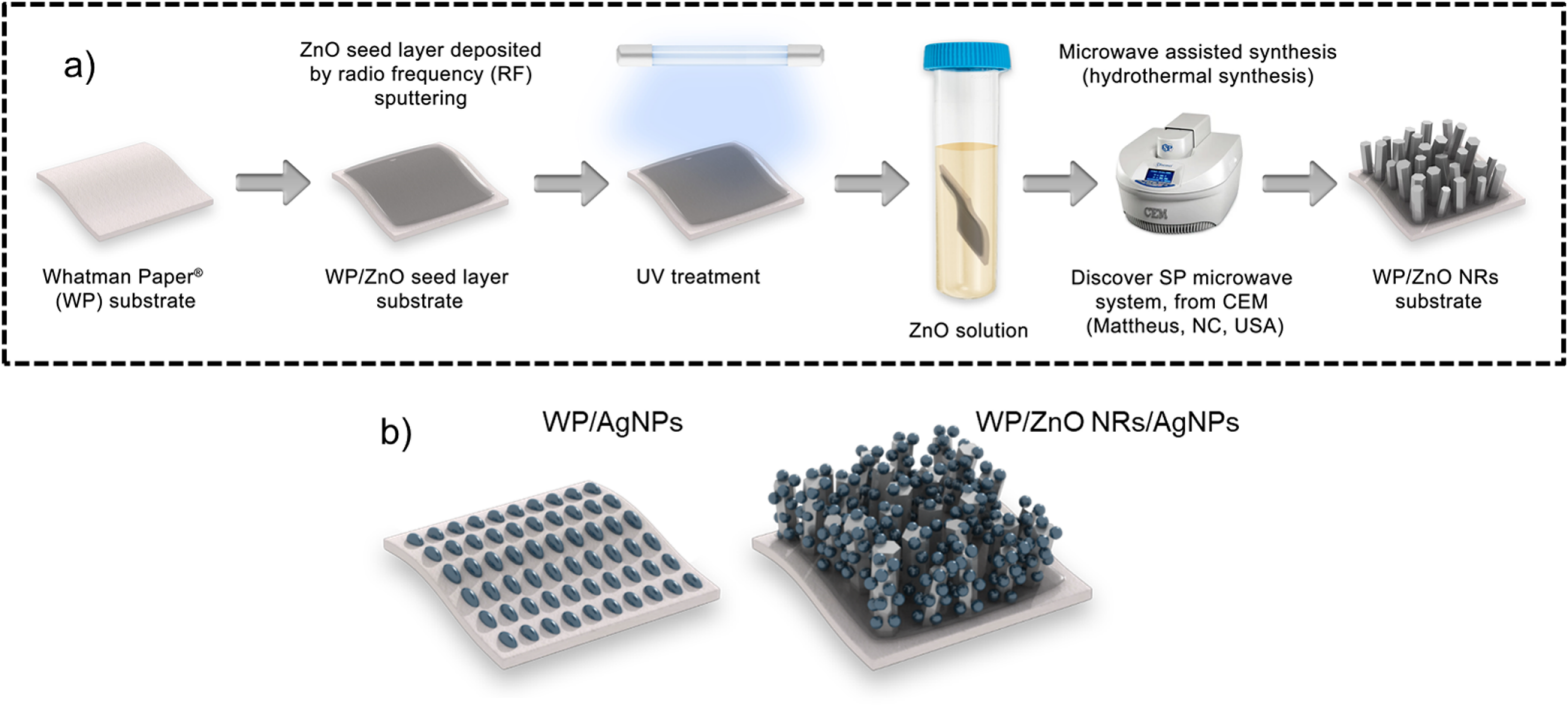
(a) Schematic of ZnO NRs hydrothermal synthesis assisted by microwave irradiation on WP substrate and (b) WP and WP/ZnO NRs substrates decorated with AgNPs.

### 2.3 Preparation of SERS Substrates

Silver nanoparticles (AgNPs) were deposited on WP and WP/ZnO NRs substrates, as illustrated in Figure 1b. Thus, two types of SERS substrates were investigated, one without (WP/AgNPs) and the other with ZnO NRs (WP/ZnO NRs/AgNPs). AgNPs were formed by depositing thin layers of silver directly onto the substrates using a home-built electron beam evaporation system.^21^ High-purity silver (99.99%) was used as the source material and placed in a graphite crucible. The substrate was left under vacuum for 12 h, until it reached a minimum of 10^–7^ Torr to minimize contamination and oxidation. The system was then heated up to 150 °C and left at that temperature for 2 h to stabilize. The substrate temperature was maintained at 150 °C throughout the deposition to promote surface diffusion and controlled nanoparticle formation, resulting in a mass-equivalent thickness of 6 nm of Ag at a controlled rate of 0.07 nm s^–1^, monitored using a calibrated quartz crystal microbalance (QCM). The combination of low deposition thickness and elevated substrate temperature facilitated the self-assembly of discrete Ag nanoparticles via Volmer–Weber (island) growth mode, rather than forming a continuous film.^42^ After the deposition, the system was left to cool for 24 h to reach room temperature, before removing the sample.

R6G was used to optimize both SERS substrates (WP/AgNPs and WP/ZnO NRs/AgNPs). Different solutions of R6G were prepared using Milli-Q water as solvent. Samples were prepared by dropping 10 μL of R6G solutions with varying concentrations (10^–4^, 10^–5^, 10^–6^, 10^–7^, 10^–8^ and 10^–9^ mol/L) onto the SERS substrates. For pesticide detection, different concentrations of TBZ (10^–3^, 10^–4^, 10^–5^, 10^–6^, 10^–7^, 10^–8^, 10^–9^, and 10^–10^ mol/L) were prepared and dropped (10 μL) onto the SERS substrates. To assess the reliability and reproducibility of the results, five different substrates were tested for each concentration of the analyte. TBZ and CBZ are poorly water-soluble; therefore, a stock solution (10^–2^ mol/L) was prepared using methanol and ethanol, respectively, and diluted in ultrapure water. CBZ was mixed with TBZ in equal proportions to assess the selectivity of the SERS substrates.

### 2.4 Characterizations

Scanning electron microscope (SEM) images of the WP, WP/AgNPs and WP/ZnO NRs/AgNPs substrates were acquired using a Regulus 8220 Scanning Electron Microscope. The dimensions of the ZnO NRs and AgNPs were estimated using ImageJ software, where three nanostructures were measured.

X-ray diffraction (XRD) was used to study the crystallinity of ZnO NRs and the ZnO NRs/AgNPs structures using PANalytical’s X’Pert PRO MRD X-ray diffractometer (PANalytical B.V., Almelo, The Netherlands), with a monochromatic Cu Kα radiation source with a wavelength of 1.540598 Å. XRD measurements were recorded with a scanning step size of 0.033° from 20° to 80° (2θ) using an X’Celerator 1D detector.

The WP/AgNPs and WP/ZnO NRs/AgNPs substrates were also evaluated by X-ray photoelectron spectroscopy (XPS). XPS measurements were carried out in a Kratos Axis Supra spectrometer using monochromated Al Kα radiation (1486.6 eV). The detail spectra were measured with a pass energy of 10 eV. Data analysis was done with CasaXPS, using the relative sensitivity factors provided for the instrument.

Raman measurements were carried out with a Renishaw inVia Qontor confocal Raman microscope equipped with a Renishaw Centrus 2957T3 detector and a 633 nm laser operating at 50 mW and diffraction grating with 1800 l/mm. All the spectra were recorded with 1 s of integration time and 5 accumulations. The laser beam was focused with a 20× Olympus objective lens. R6G and TBZ pesticides were chosen as analytes to investigate the performance of the WP/AgNPs and WP/ZnO NRs/AgNPs substrates for SERS detection. The SERS substrates were prepared by dropping 10 μL of analytes on a substrate. All samples were allowed to dry at room temperature. Data is expressed as mean ± standard deviation from at least five independent experiments (five distinct substrates).

The 633 nm laser was chosen based on a series of preliminary tests, in which this wavelength provided the best balance between Raman signal intensity and minimal fluorescence interference. Tests with the 532 nm laser resulted in strong fluorescence, likely due to the excitation of impurities or functional groups present in the cellulose structure of the paper substrate. In comparison, the 785 nm laser did not produce detectable signals. In this way, the 633 nm laser was the most suitable choice for the WP-based substrates.

## 3 Results and Discussion

### 3.1 Characterization and Optimization of WP-Based SERS Substrates

The morphology of the WP substrates can be seen in Figure S1a,b. Our team members have already conducted a detailed study on the characteristics of the WP substrate. According to Pimentel et al.,^40^ the WP has a surface with a root-mean-square (RMS) roughness of 12.6 μm and no calcium carbonate (CaCO_3_) or other contaminants. Furthermore, the WP substrates exhibit a high density of cylindrical-shaped cellulose fibers intertwined. Figure 2a,b show the morphology of the WP substrates with 6 nm of AgNPs where the complete coverage of the cellulose fibers with the AgNPs is perceptible. The morphology of the AgNPs deposited onto a silicon substrate is shown in Figure S1c,d, and was used as a reference for a more accurate analysis of their size. The average diameter of the AgNPs is ∼55 nm. Figure 2c,d show the morphologies of the WP substrates with the ZnO seed layer. The deposition of the ZnO seed layer onto the substrates precedes the ZnO NRs growth process, as this layer decreases the interfacial mismatch between the metal oxide and the substrate by providing nucleation sites and consequently reducing the thermodynamic barrier to crystallization.^43^ Before the hydrothermal synthesis, it is vital to expose the seed layer to a UV treatment to stimulate the nanostructures’ growth along the *c*-axis (perpendicular to the surface of the substrate). This occurs because exposing the ZnO seed layer to UV light will decompose the adsorbed O_2_ on the surface, making the surface more polar, with a zinc terminal plane (0001), which will promote the growth of ZnO nanorods by the hydrothermal synthesis method assisted by microwave irradiation.^40,41^

**Figure 2:**
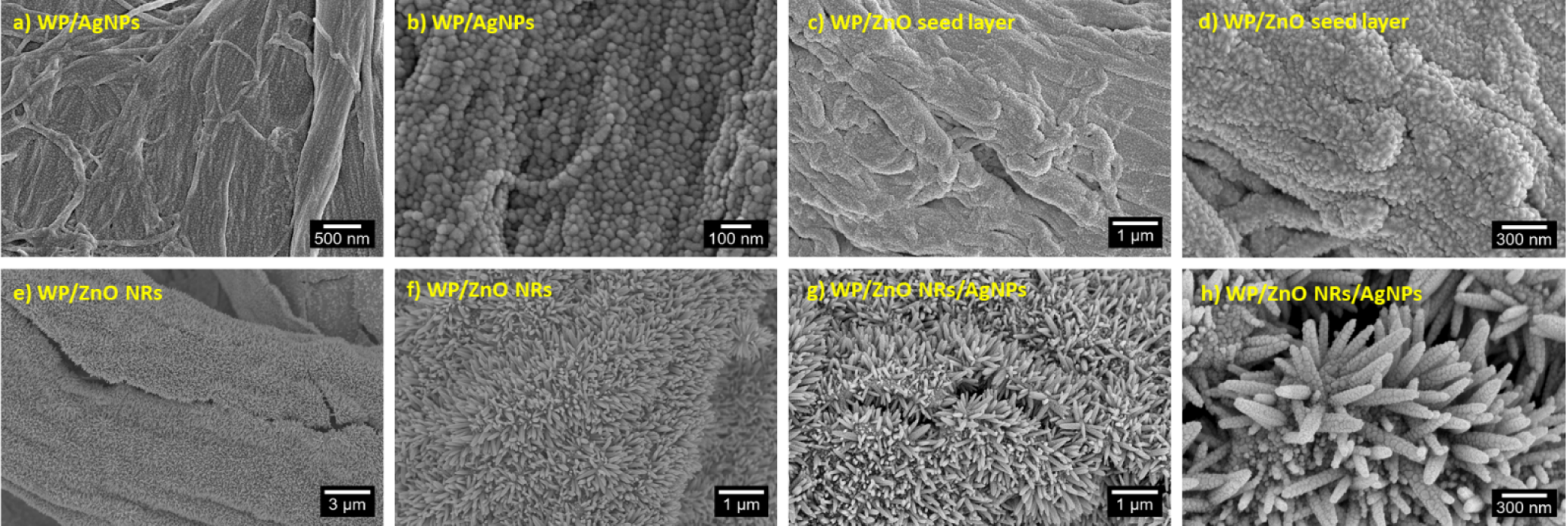
SEM images of: (a) and (b) WP substrates with silver nanoparticles (WP/AgNPs); (c) and (d) WP substrates with zinc oxide (ZnO) seed layer; (e) and (f) WP substrates with ZnO nanorods (WP/ZnO NRs); (g) and (h) ZnO NRs decorated with AgNPs (WP/ZnO NRs/AgNPs).

Figure 2e,f show the morphology of the ZnO NRs, in which a homogeneous distribution of nanorods oriented perpendicularly to the surface of the fibers can be observed along the cellulose fiber’s length. Such morphology indicates that the growth process was successful, resulting in hexagonal-shaped nanorods, nearly aligned, homogeneous, with high density across the entire substrate, and with an average length of ∼300 nm. ZnO NRs contribute to increasing the surface area of the substrate and can also mitigate the negative effects of irregular paper morphology due to its high porosity. It is worth mentioning that the growth of ZnO NRs occurs on a ZnO seed layer (200 nm) deposited on the paper. Thus, in addition to promoting the oriented growth of nanorods, this seed layer can also help to reduce the surface irregularities inherent to the paper substrate. Figure 2g,h show the morphologies of the ZnO NRs coated with AgNPs, demonstrating the uniform coating of the nanorods. The profile of ZnO NRs and ZnO NRs/AgNPs on the paper substrates is shown in Figure S1e,f. A relevant feature that should be highlighted is the presence of well-defined individual boundaries between the nanoparticles, as shown in Figures 2h and S1g. These boundaries indicate that the nanoparticles have not merged into a continuous film, thereby preserving nanoscale gaps between them. Such gaps are essential for the formation of “hot spots”, which promote localized electromagnetic field enhancement and, consequently, significantly improve the SERS signal. Differences in the size of AgNPs can be observed in the different substrates (silicon wafer, WP, and WP/ZnO NRs), which may be attributed to variations in surface properties and morphology. The silicon wafer, with its smooth and homogeneous surface, favors the formation of uniform nanoparticles with consistent size. In contrast, the highly porous and fibrous nature of the WP substrate can lead to greater variability in AgNP size. For the WP/ZnO NRs substrates, an irregular size distribution of AgNPs was observed, likely due to the nanorod geometry, which promotes heterogeneous nucleation and growth of the nanoparticles.

Figure 3a,b and e show the X-ray diffractograms of the WP, WP/ZnO seed layer, WP/ZnO NRs, and WP/ZnO NRs/AgNPs substrates. Crystalline peaks (highlighted in pink) were observed for all samples, with the XRD pattern of the cellulose appearing in each case. The WP/ZnO seed layer substrate displays diffraction planes (11̅0), (110), (200), and (002) at 2θ values of 14.8°, 16.58°, 22.84°, indicative of the polycrystalline nature of the paper substrate, and 34.36°, associated with the polycrystalline structure of the ZnO seed layer film.^44^ ZnO NRs exhibit a hexagonal wurtzite structure, as evidenced by seven diffraction peaks (highlighted in black) at 2θ angles of 31.89°, 34.36°, 36.37°, 47.66°, 56.69°, 62.96°, and 67.97°, corresponding to crystal planes of (100), (002), (101), (102), (110), (103), and (112), respectively^45^ according to the ICDD card 036-1451. The higher peak intensity of the (002) crystal plane, when in comparison with the (100) and (101) peaks suggests that ZnO NRs preferentially grow perpendicular to the surface of the substrate fibers, consistent with SEM images in Figure 2e,**f**. As can be seen, the WP substrate with ZnO NRs/AgNPs did not show significant differences compared to the substrate with only ZnO NRs, indicating the decoration of the metal oxide nanostructures.

**Figure 3:**
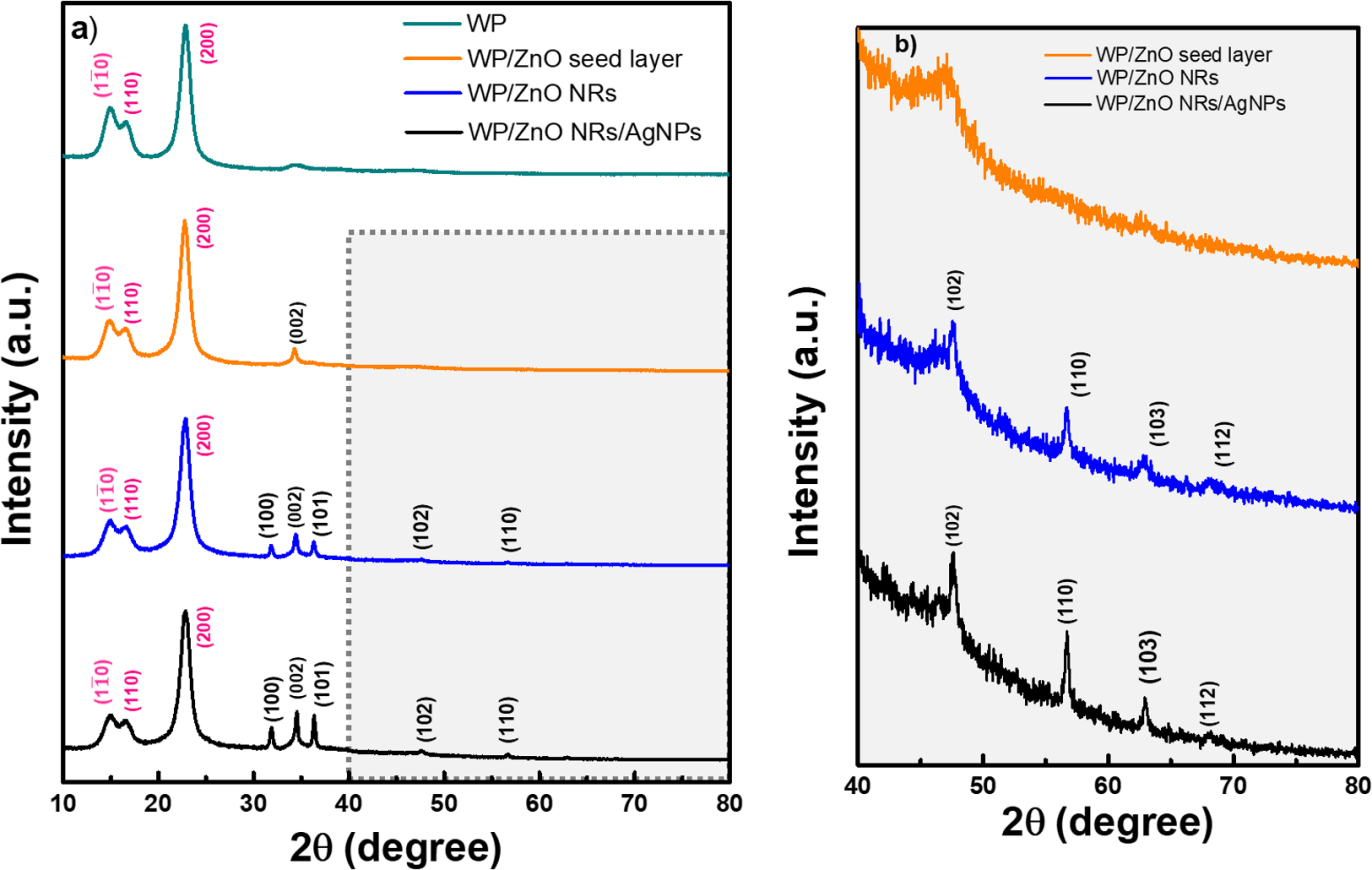
(a) X-ray diffractogram of the WP, WP/ZnO seed layer, WP/ZnO NRs and WP/ZnO NRs/AgNPs. (b) Zoom in the region between 40° and 80° of the XRD. The planes marked in pink refer to the cellulose of the WP substrate, and those marked in black refer to ZnO.

Figure 4 shows the XPS spectra of ZnO NRs/AgNPs. The results shown in Figure 4a confirm the presence of zinc (Zn), oxygen (O), and silver (Ag) in the ZnO/Ag sample, whereas only zinc and oxygen are present in the ZnO sample (besides adventitious carbon, respectively). Figure 4b displays the high-resolution XPS spectra of Zn 2p. The Zn 2p_3/2_ binding energy is located at 1022.45 eV indicating that the zinc within the structure is in the form of Zn^2+^.^46^ Compared with pristine ZnO, there is no binding energy shift in ZnO NRs/AgNPs. In the high-resolution XPS spectra of Ag 3d (Figure 4c), the Ag 3d_3/2_ binding energy is found at 368.37 eV and corresponds to metallic silver.^47^ The Ag MNN Auger emission confirms this assignment (Figure S2). The O 1s spectra are shown in Figure 4d. The 531.1 and 531.2 eV peaks, respectively, can be attributed to lattice oxygen in ZnO NRs. Two broader peaks at higher binding energies can be assigned to the surface OH-groups and adsorbed water as well as organic species.^48^ No significant changes in relative intensities of these high-energy O 1s components can be observed for the ZnO/Ag structures, compared to the ZnO.

**Figure 4:**
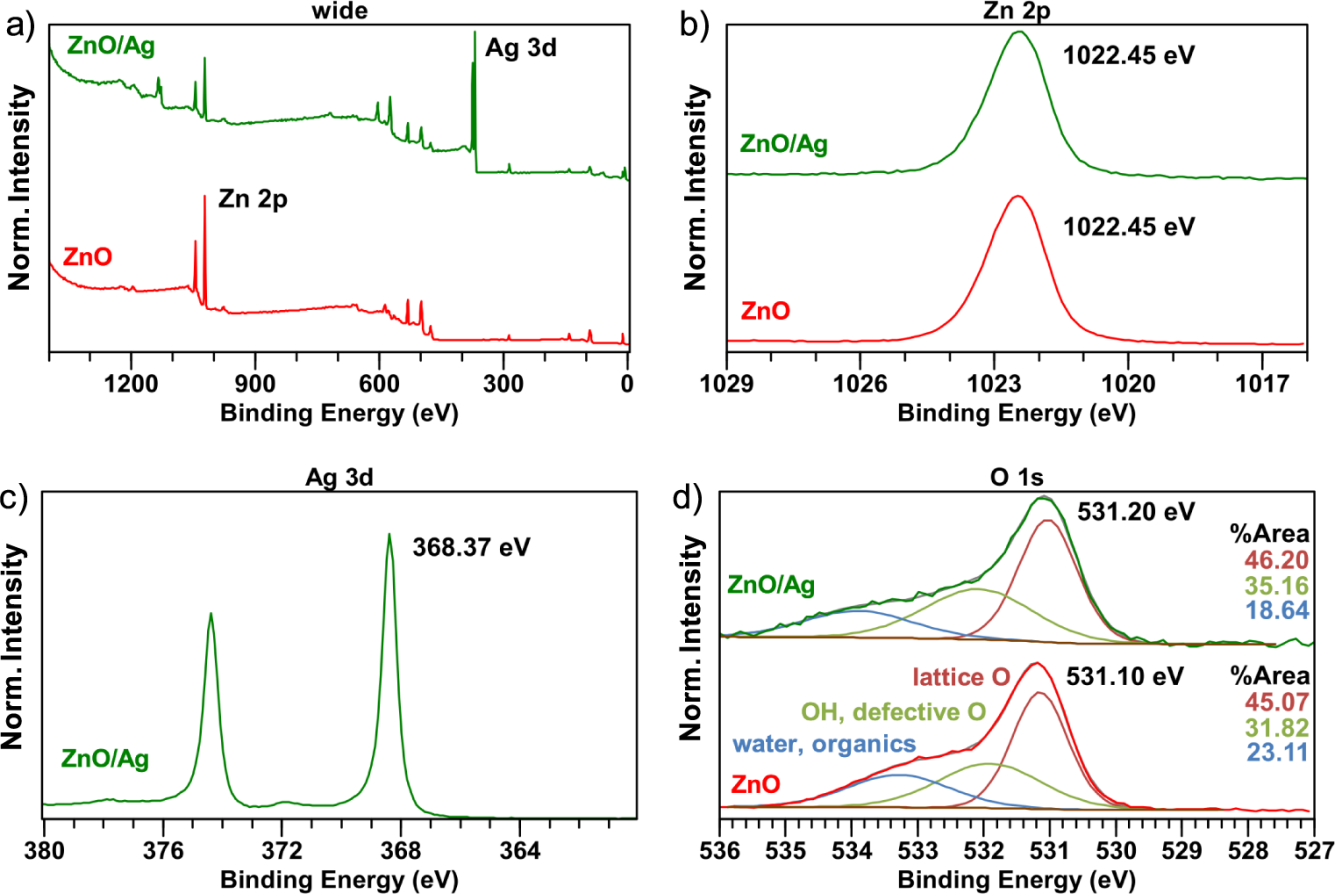
(a) XPS survey scans of ZnO/Ag and high-resolution XPS spectra of (b) Zn 2p, (c) Ag 3d, and (d) deconvoluted O 1s.

### 3.2 Performance of WP/AgNPs and WP/ZnO NRs/AgNPs Substrates Using R6G Probe

The Raman spectra of the WP, WP/AgNPs, WP/ZnO seed layer, WP/ZnO NRs, and WP/ZnO NRs/AgNPs substrates are shown in Figure S3. These spectra were obtained for comparative analysis with SERS spectra in the presence of R6G. Note that none of the bands present in the Raman spectra of the substrates (Figure S3) are present in the SERS spectra of the analytes. The optimization of WP/AgNPs substrates (without ZnO NRs) and WP/ZnO NRs/AgNPs was conducted at varying concentrations of R6G (10^–4^ to 10^– 9^ mol/L), with 10 μL of solution dropped on the substrates and subsequently dried at room temperature. Figure 5 shows the SERS spectra of R6G molecules with main bands at 611 (C–C–C ring in-plane bending), 772 (C–H out-of-plane bending), 1127 and 1183 cm^–1^ (C–H in-plane bending). The peaks associated with aromatic stretching vibrations are also present at 1311, 1360, 1510, 1573, and 1648 cm^–1^.^49^ WP/AgNPs substrate achieved a SERS signal from R6G up to a 1 × 10^–7^ mol/L concentration. On the other hand, the WP/ZnO NRs/AgNPs substrate achieved the R6G signal at an order of magnitude lower (1 × 10^–8^ mol/L). The variation of the SERS signal intensity as a function of R6G concentration on both substrates is shown in Figure S4a,b.

**Figure 5:**
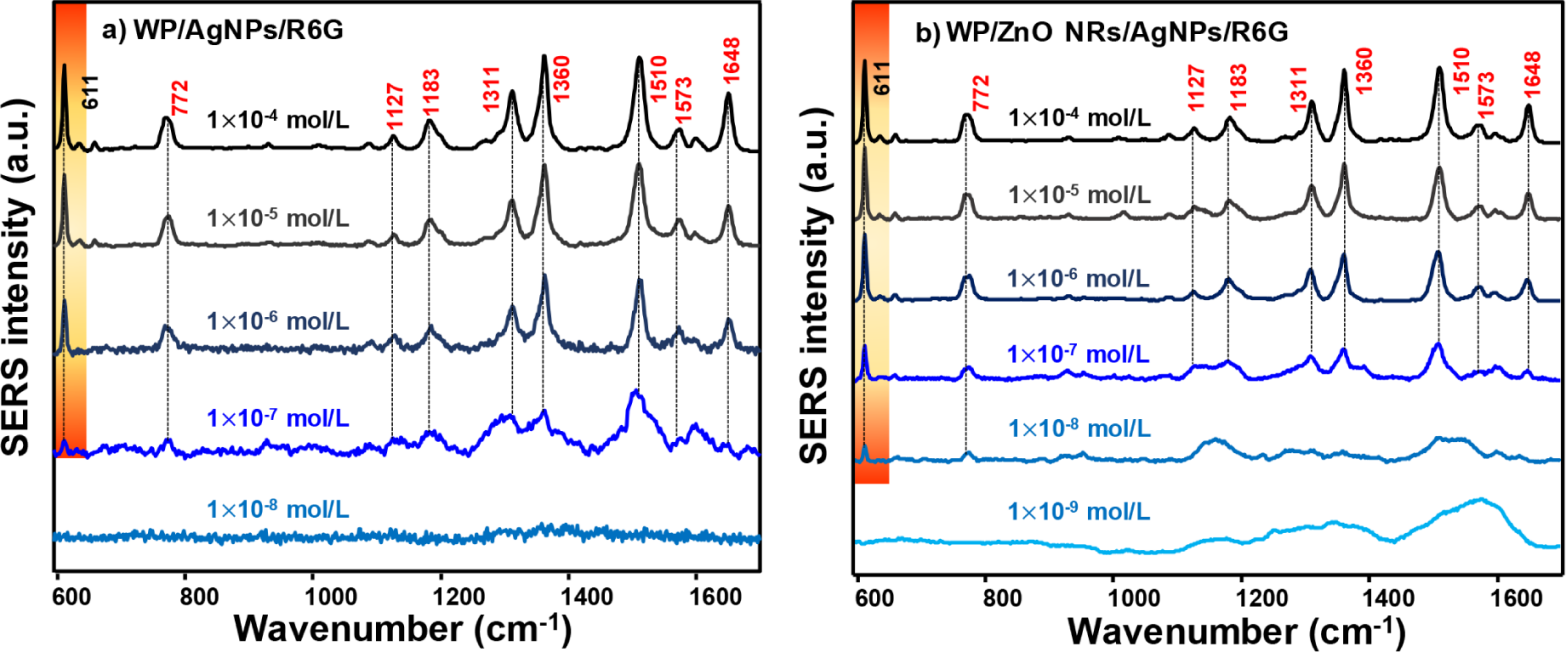
SERS spectra of R6G at different concentrations on (a) WP/AgNPs and (b) WP/ZnO NRs/AgNPs substrates. The 611 cm^–1^ band (highlighted) was used as a reference for SERS analysis of R6G. The spectra were recorded using a 633 nm laser line and plotted with baseline correction.

Five distinct substrates were characterized for each analyte concentration to ensure the reliability and reproducibility of the results. The obtained spectra were consistent across the different samples, demonstrating good reproducibility of the SERS response. These results reinforce the potential of the paper substrates as effective platforms for SERS-based detection. The bar graph in Figure 6a shows the average intensities for the 611 cm^–1^ (C–C–C ring in-plane bending) band of the R6G SERS spectrum in both substrates (WP/AgNPs and WP/ZnO NRs/AgNPs) for different concentrations of R6G. At all concentrations, the substrates containing ZnO NRs were more effective in detecting the R6G molecule, exhibiting a higher SERS signal. This increase is attributed to the larger surface area the ZnO NRs provide. The enhancement factor (EF) was estimated to infer the SERS performance of the substrates as follows:

$$EF∼\frac{\frac{I_{SERS}}{\left[N_{SERS}\right]}}{\frac{I_{RS}}{\left[N_{RS}\right]}}=\frac{I_{SERS}}{I_{RS}}\times \frac{N_{RS}}{N_{SERS}}$$

**Figure 6:**
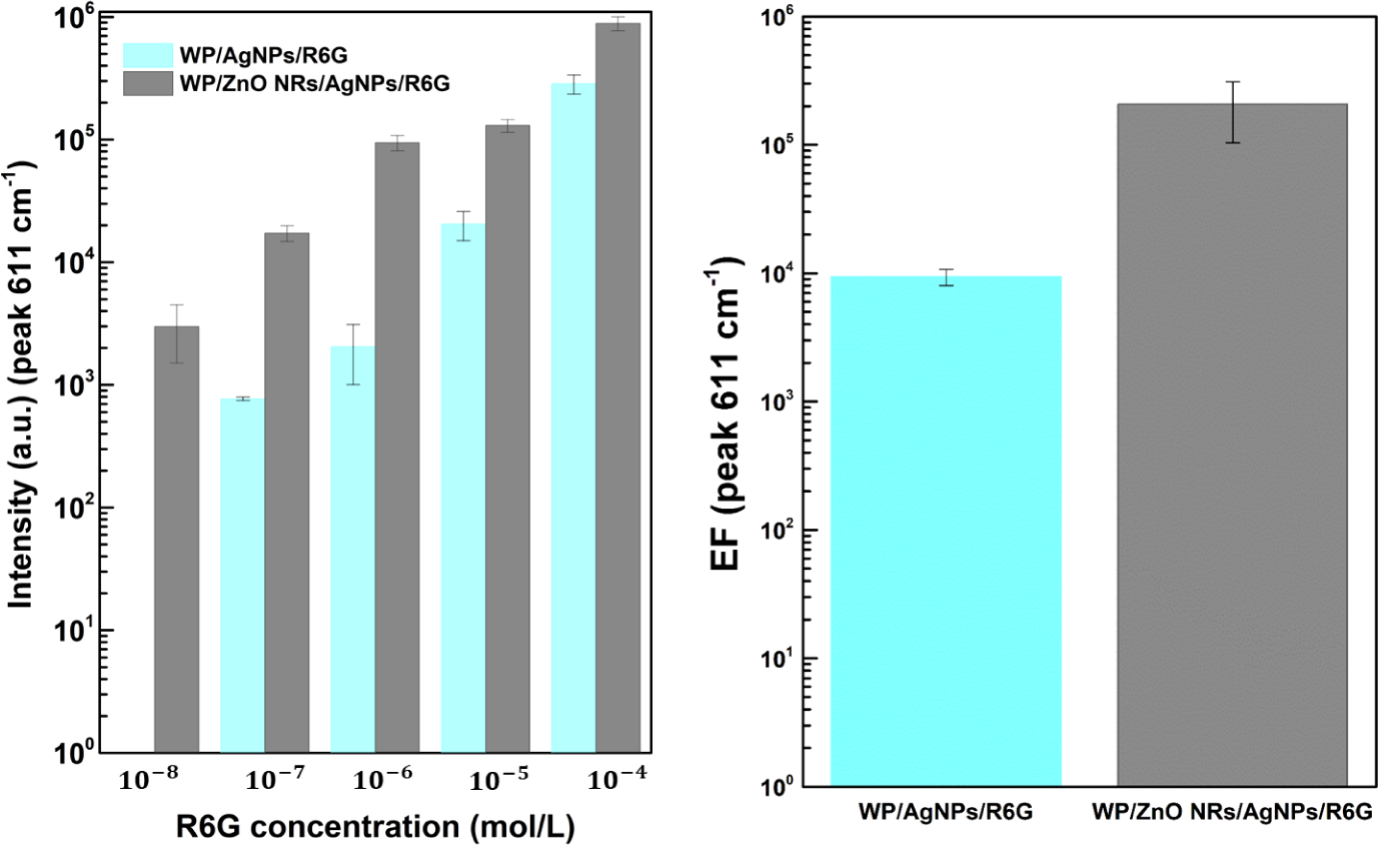
(a) Plot of the intensities of the 611 cm^–1^ peak as a function of R6G concentration and (b) EF graph for WP/AgNPs and WP/ZnO NRs/AgNPs substrates.

The EF in SERS is a measure of the Raman signal intensity of several molecules adsorbed on a metal surface (*I*_*SERS*_) compared to the Raman signal intensity of the same number of molecules (*I*_*RS*_). Therefore, the EF calculation involves the $\frac{I_{SERS}}{I_{RS}}$ ratio obtained under the same experimental conditions. However, it is essential to emphasize that the precise calculation of the EF in SERS is a complex matter since several factors can influence the results, such as the orientation of the molecule adsorbed on the surface and the coverage density of the metallic surface.^16^ The estimated EF for WP/AgNPs and WP/ZnO NRs/AgNPs were approximately 9.3 × 10^3^ and 2.1 × 10^5^, respectively, as shown in Figure 6b. Further details on the EF calculation are provided in the Supporting Information (last page). The LOD was estimated based on the signal-to-noise ratio (S/N). The concept underlying the S/N approach involves defining the LOD as the analyte concentration that produces a signal (peak or plateau) significantly more intense than the noise, which is the signal recorded in the absence of the analyte, also known as the blank signal. This method is commonly employed, assuming that noise can be accurately estimated. Setting the LOD at an S/N ratio of 3.0 allows for demonstrating the presence of the analyte in the test sample, with a probability exceeding 99%.^50^ The LOD values for R6G were approximately 4.8 × 10^–8^ and 4.3 × 10^–9^ mol/L for WP/AgNPs and WP/ZnO NRs/AgNPs, respectively. These values are particularly significant when considering the type of substrate used, in this case, WP substrate. Due to its high porosity and irregular morphology, paper exhibits distinct characteristics in terms of functionalization with semiconductor nanostructures compared to conventional substrates such as silicon. Despite the potential limitations imposed by the inherent morphology of paper such as porosity, the results presented here are remarkable and highlight the effective functionalization and potential of flexible, portable and sustainable SERS substrates. This underscores paper-based platforms as a promising alternative for low-cost, environmentally friendly sensing applications.

As mentioned earlier, SERS enhancement follows three mechanisms: (i) enhancement of the local electromagnetic field provided by MNPs, (ii) increase in the surface area provided by semiconductor nanostructures, and (iii) chemical enhancement supported by semiconductors caused by charge transfer between the metal and the semiconductor.^51^ Generally, the Raman enhancement of the electromagnetic mechanism is much higher than that of the chemical enhancement mechanism.^52,53^ Due to electromagnetic enhancement, various SERS substrates based on MNPs have demonstrated prominent SERS properties. However, several MNPs nanostructures suffer significant fabrication difficulties, high cost, and stability issues, severely limiting their applications.^54^ An efficient strategy to overcome these drawbacks is the introduction of semiconductor materials to form substrates with semiconductor/MNPs heterostructures.^55,56^ Thus, ZnO NRs/AgNPs substrates exhibit superior SERS activities because they can provide a high density of ″hot spots″ for the electromagnetic enhancement mechanism and an adequate charge transfer pathway between semiconductors and noble metals for the chemical enhancement mechanism.

Regarding the superior performance of ZnO NRs/AgNPs substrates in the detection of R6G, according to Chen et al.^48^ the conduction band (CB) and valence band (VB) of ZnO are −4.19 and −7.39 eV (with a band gap of 3.2 eV), respectively. The CB of ZnO lies between the Fermi level of AgNPs (−4.26 eV) and the lowest unoccupied molecular orbital (LUMO) level of R6G molecules (−3.4 eV), as shown in Figure S5. The energy difference between the Fermi energy level of AgNPs and the LUMO energy level of R6G is less than the energy provided by the 633 nm laser (∼1.96 eV). In other words, the energy of the exciting light (633 nm laser) is much higher than both potential energy differences (between the Fermi level of AgNPs and the CB position of ZnO and between the CB position of ZnO and the LUMO level of R6G molecules). Therefore, the electrons in the AgNPs have sufficient energy to transfer to the CB of ZnO and subsequently to the LUMO level of R6G molecules. The CB of ZnO acts as a ″bridge″ for electron transfer, increasing charge transfer and consequently enhancing the SERS signal. Furthermore, ZnO exhibits a prominent ability to provide an efficient charge transfer pathway because noble metals’ high Fermi energy level could facilitate electron transfer between metallic NPs and ZnO.^48^

### 3.3 Application of the WP/ZnO NRs-Based SERS Substrates for TBZ Pesticide Detection

The molecule structure and Raman spectrum of TBZ powder and its assignments are detailed in Figure S6a. Peaks are observed at 785, 882, 902, 991, 1016, 1281, 1460, 1582, 1595, and 1626 cm^–1^. The strong peaks at 785, 882, 902, and 1016 cm^–1^ are assigned to the bending of C–H out-of-plane of the TBZ molecule. The peaks at 1460, 1582, 1595, and 1626 cm^–1^ result from the stretching of C=N, the peak at 991 cm^–1^ is caused by the stretching of C–S, and the stretching of the total ring is associated with the peaks at 1281 and 1582 cm^–1^.^57,58^

The SERS spectra of TBZ on WP/AgNPs and WP/ZnO NRs/AgNPs substrates are shown in Figure 7. The SERS spectrum of TBZ is highlighted by the presence of the bands at 781, 883, 1009, 1576, and 1622 cm^–1^. For the WP/AgNPs substrate (Figure 7a), a strong TBZ signal is observed up to 10^–7^ mol/L; however, for the WP/ZnO NRs/AgNPs substrate, detection is precise/visible down to 10^–9^ mol/L (Figure 7b). The increase in noise at lower TBZ concentrations can be attributed to the reduced number of molecules available for adsorption on the substrate surface. This leads to a weaker SERS signal, making the background noise more pronounced than the useful signal. SERS signal intensity as a function of TBZ concentration on both substrates is shown in Figure S4c,d. The SERS spectrum of TBZ in Figure 7 is similar to those presented in the literature.^59,60^ It is worth mentioning that in our work, the bands at 781 cm^–1^ (S–C stretching) and 1009 cm^–1^ (C=N stretching) show greater SERS intensity and a decrease of intensity of bands at 1281 and 1460 cm^–1^, when compared to the spectrum of powdered TBZ. According to Oliveira et al.,^60^ this indicates that the TBZ pesticide is adsorbing on the surface of the substrates (WP/AgNPs and WP/ZnO NRs/AgNPs) via the aromatic ring that contains sulfur (S) and nitrogen (N). This adsorption configuration affects the molecule’s orientation and may result in a decrease in the Raman intensity of certain vibrational modes, such as those at 1281 and 1460 cm^–1^.

**Figure 7:**
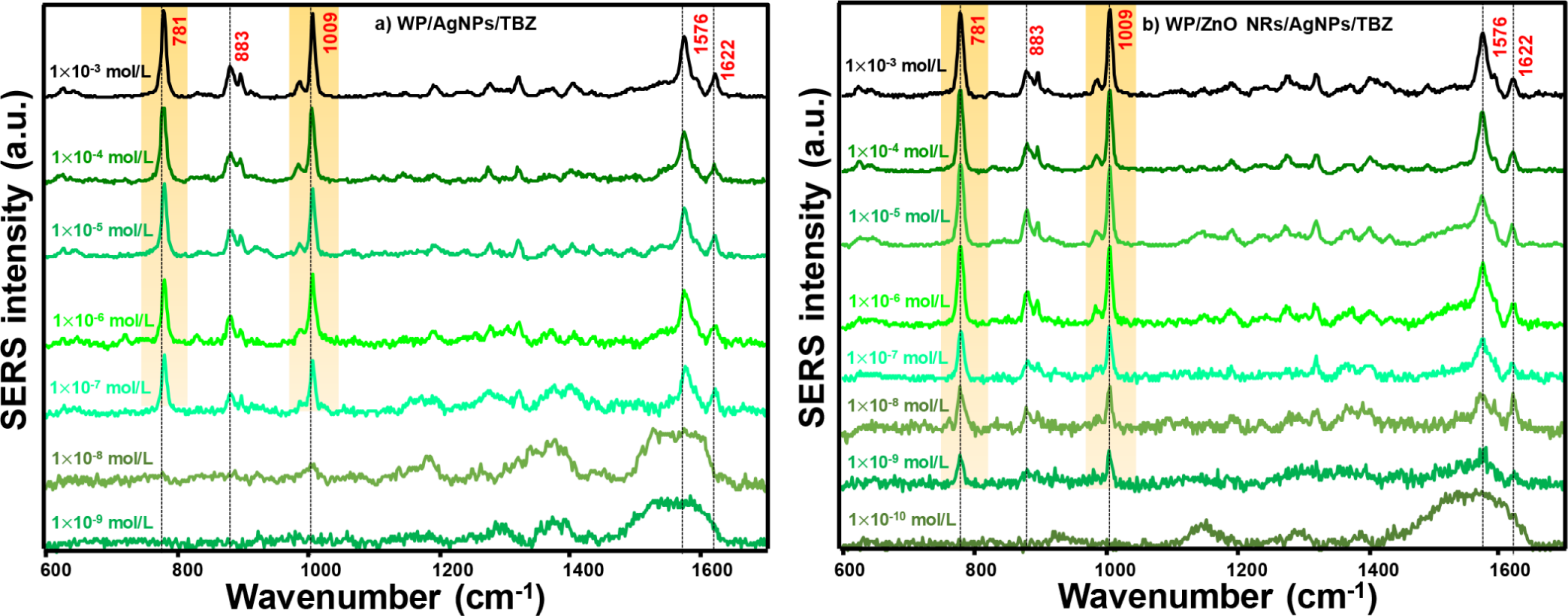
SERS spectra of TBZ in (a) WP/AgNPs and (b) WP/ZnO NRs/AgNPs substrates. The 781 and 1009 cm^–1^ band (highlighted) was used as a reference for SERS analysis of TBZ. The spectra were recorded using a 633 nm laser line and plotted with baseline correction.

Figure 8a,b show plots of intensities as a function of concentrations for the two prominent bands in the SERS spectra of TBZ at 781 (stretching of C–S) and 1009 cm^–1^ (stretching of C=N), used for fungicide detection analysis for both substrates. The substrate with only AgNPs exhibited significant variations in SERS intensity for different samples. Consequently, no distinguishable signal was obtained for concentrations below 10^–7^ mol/L (see Figure 7a). Substrates containing ZnO NRs showed fewer variations in SERS intensity compared to the substrate with only AgNPs. As shown in Figure 8b, the standard deviation of intensities as a function of concentrations is lower than the first substrate. Experimentally, the substrate with ZnO NRs proved more efficient in detection, meaning the pesticide SERS molecule signal was obtained more efficiently without extensive sample scans. Figure 8c shows a bar graph with peak intensities at 781 cm^–1^ as a function of concentration for both substrates. As mentioned above, the presence of the sulfur atom in TBZ enhances its interaction with silver, contributing to the SERS effect and resulting in lower signal intensity variation between the different substrates (see Figure 8c). However, overall, the substrates containing ZnO NRs still exhibit superior performance. This is attributed to the combined effect of the increased surface area provided by the ZnO NRs and the efficient adsorption of TBZ onto AgNPs, along with the typical electromagnetic mechanisms of SERS. These characteristics justify the enhanced response observed for the WP/ZnO NRs/AgNPs substrates compared to the WP/AgNPs substrates (without ZnO NRs). Figure 8d shows the linear variation of intensity of the 781 cm^–1^ band for the five lowest concentrations (10^–9^ to 10^–5^ mol/L) for the substrate with ZnO NRs. The EF for WP/AgNPs and WP/ZnO NRs/AgNPs substrates with TBZ were approximately 2.05 × 10^5^ and 9.8 × 10^6^, respectively, as shown in Figure 8e. Due to significant intensity variations associated with the standard deviation, the analytical LOD calculation using the signal-to-noise ratio,^50^ yielded values of 4.0 × 10^–8^ and 5.0 × 10^–10^ mol/L for the WP/AgNPs and WP/ZnO NRs/AgNPs substrates, respectively.

**Figure 8:**
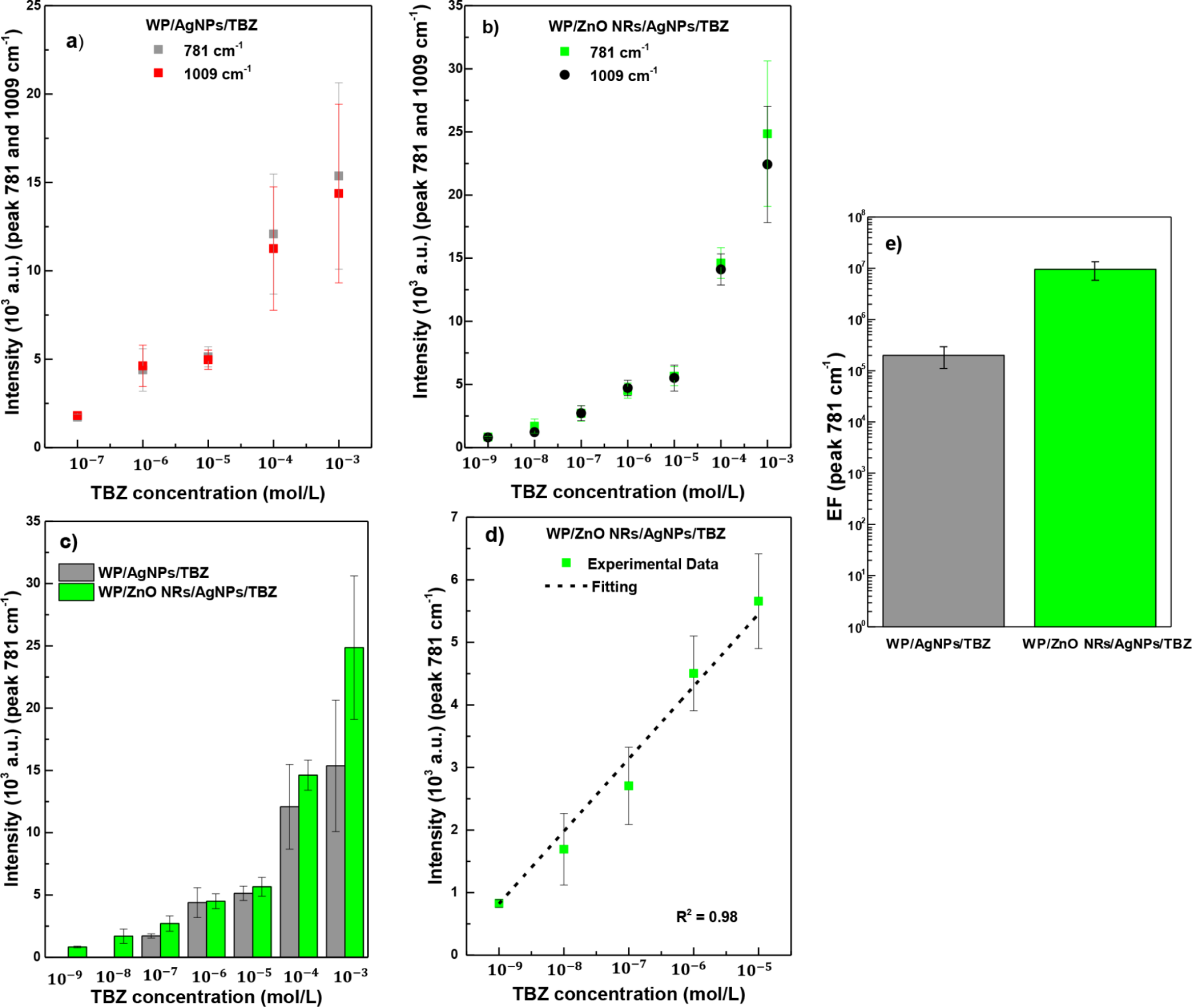
Intensities of bands at 781 and 1009 cm^–1^ as a function of TBZ concentration for (a) WP/AgNPs and (b) WP/ZnO NRs/AgNPs substrates. (c) Plot of the intensities of the 781 cm^–1^ peak of TBZ, for both substrates. (d) Analytical curve of the intensity of the 781 cm^–1^ peak as a function of concentration for WP/ZnO NRs/AgNPS substrate. (e) EF graph for both substrates.

In 2024, the European Commission Regulatory Authority (EU) 2024/1342 requested the reduction of maximum residue limits (MRLs) for TBZ in avocados and papayas. The Authority identified a potential short-term consumption concern for consumers regarding the current MRL for papayas. As a result, the limit for papayas was reduced to 0.01 mg/kg (∼5.0 × 10^–8^ mol/L in aqueous medium).^61^ This measure follows previous reductions of MRLs for this substance in mangoes and products of animal origin, which have been in force since September 2023. In Brazil, however, the permitted MRLs are sometimes higher than those adopted in Europe. For example, in the case of papayas, the acceptable MRL can reach up to 0.1 mg/kg. Considering these new limits proposed by the European Union, SERS substrates with ZnONRs/AgNPs show potential as agricultural sensors, as they can detect concentrations even lower than the regulatory limits.

Table 1 shows the performance of different SERS substrates, both solid and liquid, recently reported (2018–2024) in the literature for TBZ detection. It is worth mentioning that none of the mentioned works report the use of paper substrates with ZnO NRs. Regarding the use of Au and Ag NPs colloids, this method presents LODs comparable to those reported on paper substrates with AgNPs, indicating that the MNPs’ immobilization does not affect the SERS enhancement. The comparison of results obtained with ZnO-based SERS platforms shows that the use of this nanostructures favors the preparation of efficient SERS substrates for sensitive and selective detection of analytes, contributing to various fields ranging from fundamental research to practical applications in environmental monitoring, healthcare, food safety, and beyond.

**Table 1:** Different Substrates Referring to Literature Works from 2018 to 2024 on the Detection of TBZ, with Their Respective EF and LOD

SERS substrates	EF	LOD	References
Au colloid	-	10^–9^ mol/L	^62^
Au on SiO_2_ nanospheres	∼10^15^	∼10^–18^ mol/L	^63^
Au@Ag/PMMA/qPCR–PET film (solid substrate)	3.14 × 10^6^	7.0 × 10^–5^ mol/L	^64^
eggshell membrane coated with AuNPs	-	0.1 ppm	^65^
Ag colloid	-	13.8 ppb	^60^
Ag/nanocellulose fibers	-	0.09 ppm	^66^
Au@Ag NRs colloid	-	0.032–0.034 ppm	^59^
AgNPs on office paper	-	0.097 ppm	^67^
AgNPs on Whattman paper	2.05 × 10^5^	4.0 × 10^–8^ mol/L	this work
ZnO NRs/AgNPs on Whattman paper	9.8 × 10^6^	5.0 × 10^–10^ mol/L	this work

Figure S7 presents the SERS spectra of TBZ at a concentration of 1 × 10^–7^ mol/L, obtained after 1 and 3 months following the fabrication of WP/ZnO NRs/AgNPs substrates, to evaluate their long-term stability. It is emphasized that the stability analysis was conducted based solely on variation over time, with the substrates stored under ambient conditions (uncontrolled temperature and humidity). Even after 1 month, the substrates could detect the pesticide, displaying the main characteristic bands at 781, 1009, and 1576 cm^–1^. After 3 months, it was still possible to identify the 781 cm^–1^ band band highlighting the viability of using the developed platforms for pesticide detection even after prolonged storage, however, the remaining bands were characteristic of the substrate. These results not only demonstrate the reproducibility and stability of the substrates, but also indicate that their functionality is maintained even when stored under ambient conditions, without the need for special handling. In addition to the long-term stability test, we also compared the performance of the paper-based ZnO NRs/AgNPs substrates with a commercial Au SERS substrate. The commercial substrate exhibited inconsistent performance, even at high concentrations (1 × 10^–3^ mol/L). This result and its high cost make it impractical for in situ applications. The main limitations of commercial substrates include production variability, inconsistent performance, short lifespan (typically less than 1 month), rapid degradation, and, in many cases, specificity for only specific analytes. Therefore, investing in research to develop more efficient, sustainable, cost-effective SERS substrates is essential. Overcoming the limitations of commercial substrates broadens the applications of SERS technology, making it an even more powerful and versatile tool across various scientific and industrial fields. The results obtained with paper substrates functionalized with ZnO nanostructures are promising, demonstrating good efficiency and a longer lifespan compared to most commercially available SERS substrates.

### 3.4 Substrate Selectivity: (WP/ZnO NRs/AgNPs) in the Presence of TBZ and CBZ Pesticide

Most studies reported in the literature focus only on detecting a single pesticide at a time, or they only perform qualitative analyses without quantifying multiple pesticides. However, in agriculture, it is common to apply a mixture of two or more pesticides. This approach is adopted as a strategy to increase the effectiveness of pest control and to prevent the development of resistance by target organisms to individual pesticides. Thus, SERS substrates must be capable of detecting such mixtures. To assess the efficiency of the WP/ZnO NRs/AgNPs, we conducted tests by mixing TBZ and CBZ to evaluate their SERS response. CBZ and TBZ are two of the most widely used benzimidazole fungicides in modern agriculture, mainly for preharvest and postharvest protection against fungal diseases in fruits and vegetables.^39^ Although CBZ and TBZ molecules have similar molecular structures with the same benzimidazole group (see Figure S6), the fungicide TBZ contains a sulfur atom that is generally more active in SERS than the oxygen atoms present in CBZ.

Figure 9 presents the SERS spectra of TBZ and CBZ on WP/ZnO NRs/AgNPs and of the pesticide mixture (TBZ+CBZ), both at 1 × 10^–5^ mol/L. The SERS spectrum of the CBZ is characterized by bands at 631 (ring stretching and C–C bending), 735 (C–C bending and C–O–CH_3_ bending), 1009 (C–N bending, C–C bending and C–O–CH_3_ stretching), 1228 and 1272 (C–H bending and N–H bending), 1460 (C–H bending and N–H bending) and 1520 cm^–1^ (N–H bending and C–N stretch)^68^ (see Figure 9b). The characteristic bands in the SERS spectrum of TBZ are at 781, 1009, 1576, and 1622 cm^–1^, as shown in Figure 9a. Figure 9c shows the overlapping band at 1009 cm^–1^ referring to the same groups (benzimidazole) present in CBZ and TBZ insecticides. However, the two dotted circled regions show the characteristic bands of CBZ, as mentioned above, at 1228, 1272, 1460, and 1520 cm^– 1^, which are also present in the mixture with TBZ. Note that these CBZ bands refer to the adsorption of nitrogen (N) atoms close to the silver surface. The work of Furini et al.^69^ demonstrates that this region is responsible for the adsorption of the fungicide on the metal surface. However, a higher noise level is observed in the CBZ spectrum, which may be related to the low adsorption efficiency of the molecules on the silver surface and/or a poor match between the excitation wavelength and the molecular absorption characteristics of the analyte. Hassan et al.^70^ reported the detection of the mixture of TBZ and CBZ using label-free anisotropic bimetallic hollow Au/Ag nanostars (HAu/Ag NS) SERS substrate in combination with DFT and SPE. However, to the best of the authors’ knowledge, the detection of both pesticides on paper-based SERS substrates has not yet been reported in the literature.

**Figure 9:**
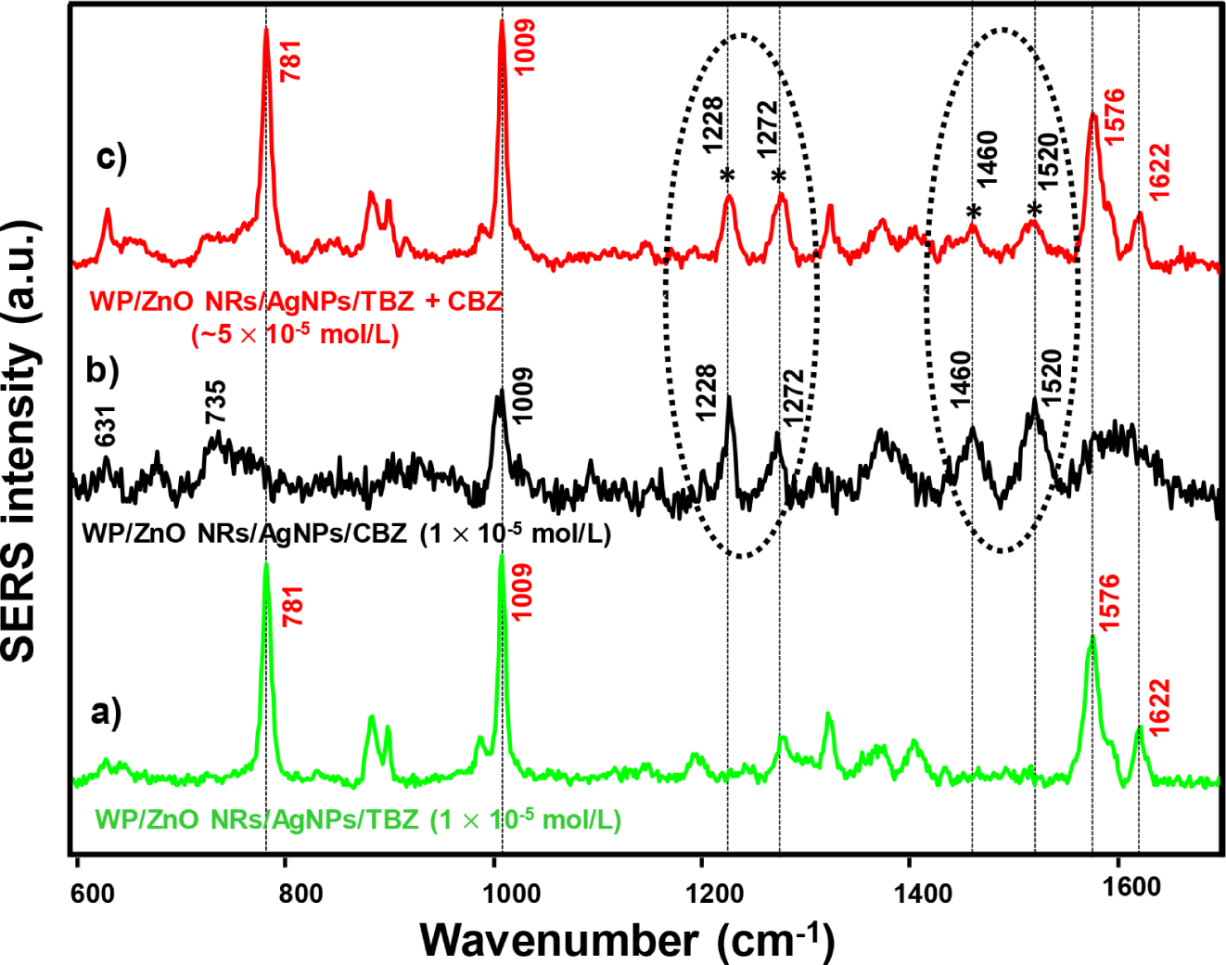
SERS spectra of (a) TBZ, (b) CBZ, and (c) a mixture of TBZ and CBZ (1:1) on WP/ZnO NRs/AgNPs substrates. The spectrum of the mixture (in red) shows the characteristic peaks of TBZ and CBZ (highlighted in dotted circles). The spectra were recorded using 633 nm laser line and plotted with baseline correction.

Figure S8a shows the SERS mapping of WP/ZnO NRs/AgNPs with the pesticide mixture (TBZ + CBZ). The SERS mapping of a specific substrate area can provide more detailed information about the distribution of the analyte of interest over the sample, thus allowing for a more thorough analysis of the results. The mapping was done over a random cellulose fiber of the WP substrate in an area of 12 μm × 25 μm, total of 950 spectra. The bands used as references for mapping TBZ and CBZ were 781 and 1228 cm^–1^, respectively. These bands were chosen because they are characteristic of each pesticide. TBZ (red map) shows a significantly higher SERS intensity compared to CBZ (blue map), which is consistent with what we observed in Figure 9. TBZ exhibits better distribution over fiber of the substrate with high intensity in most of the mapped area; however, we note the absence of CBZ in some regions. It is worth noting that due to the porous and fibrous nature of the paper substrate, it is more difficult to focus the laser accurately around the edges of the fibers. Thus, the SERS signal tends to be more intense along the paper fibers and weaker at the edges or in porous regions. Overall, the SERS mapping reinforces the capability of the paper substrate with ZnO NRs/AgNPs to detect the pesticide mixture (TBZ + CBZ). The SERS mapping of two different areas of the same substrate is shown in Figure S8b,c. The maps exhibit good reproducibility for the detection of the pesticide mixture and are consistent with what we discussed above.

Using paper as a platform for SERS substrates offers several advantages that make it highly attractive for analytical applications. It is a sustainable, widely available, lightweight material easily transported and highly adaptable to different surfaces. In this study, the substrates were designed for disposable, single-use applications; therefore, they cannot be reused once exposed to pesticides due to contamination. Nevertheless, this characteristic aligns with conducting rapid and targeted analyses, making them an efficient and cost-effective alternative to conventional SERS substrates. Tests to detect the mixture of TBZ and CBZ in WP/ZnO NRs substrate are preliminary, however, promising. Further studies are needed to evaluate the detection limit of the mixture on such substrates. It is worth mentioning that the developed SERS substrate (WP/ZnO NRs/AgNPs) is not only capable of detecting a single pesticide as is commonly found in the literature, but it also proved to be selective for the possible study of a mixture of the TBZ and CBZ pesticides. The reported results open perspectives for application in citrus cultivation systems, where these insecticides are widely used as a mixture.

## 4 Conclusions

In summary, we investigated the functionalization of Whatman no 1 paper with zinc oxide nanorods (ZnO NRs) and silver nanoparticles (AgNPs) for application as SERS substrates aimed at detecting the pesticide thiabendazole (TBZ). All substrates containing ZnO NRs/AgNPs exhibited superior SERS performance compared to substrates with only AgNPs. The limit of detection of the TBZ using only AgNPs and ZnO NRs/AgNPs were 4.0 × 10^–8^ and 5.0 × 10^–10^ mol/L, respectively, and the enhancement factors were 2.0 × 10^5^ and 9.8 × 10^6^, respectively. The paper substrates with ZnO NRs/AgNPs also demonstrated good long-term stability and selectivity for detecting pesticide mixtures. These results open the way for advancing paper-based substrates designed for the simultaneous detection of multiple environmental contaminants. Furthermore, they highlight the potential of paper as a versatile substrate and open new perspectives for exploring alternative materials such as wood or cellulose-derived structures, investigating different ZnO nanostructure fabrication methods, and expanding applications to the detection of biological targets, pesticides, and other analytes of environmental and agricultural relevance.

## Supplementary Material



## Notes

SEM images of the WP substrate, silicon wafer/AgNPs, and the profile of ZnO NRs and ZnO NRs/AgNPs; Ag MNN Auger emission; Raman spectra of the WP, WP/AgNPs, WP/ZnO seed layer, WP/ZnO NRs, and WP/ZnO NRs/AgNPs substrates; SERS intensities of R6G and TBZ at different concentrations; diagram of the charge transfer process for R6G; molecular structures and Raman spectra of TBZ and CBZ; SERS spectra of TBZ over time; SERS mapping of the TBZ and CBZ mixture; EF calculation (PDF)
