# Supplementary material for: ZnO Nanorod/Ag Nanoparticle—Functionalized Paper Substrate for Sensitive SERS Detection of Environmental Contaminants
Source: ACS Appl Nano Mater. 2025 May 14;8(20):10434–47. doi: 10.1021/acsanm.5c01103 (PMC12104969; doi:10.1021/acsanm.5c01103)
Supplement: Supplementary file 1 [file an5c01103_si_001.pdf]

Supporting Information

ZnO Nanorod/Ag Nanoparticle - Functionalized Paper Substrate for Sensitive SERS Detection of Environmental Contaminants

Maíza Ozório[1,2], Ana Pimentel[2], Maria Morais[2], Mariana Cortinhal[2], Rafael Jesus Gonçalves Rubira[3], Tatiana Aparecida Oliveira[1], Jonas Deuermeier[2], Hugo Águas[2], Luís M. N. Pereira[2], Carlos José Leopoldo Constantino[1] and Rodrigo Martins[2]

[1]São Paulo State University – UNESP, School of Science and Technology (FCT), Department of Physics, Presidente Prudente, SP, 19060-900, Brazil

[2]i3N/CENIMAT, Department of Materials Science, NOVA School of Science and Technology and CEMOP/UNINOVA, Campus de Caparica, 2829-516, Caparica Portugal

[3]Sao Paulo State University – UNESP, Institute of Geosciences and Exact Sciences (IGCE), Physics Department, Rio Claro, SP, 13506-900, Brazil
*e-mail: maiza.ozorio@unesp.br

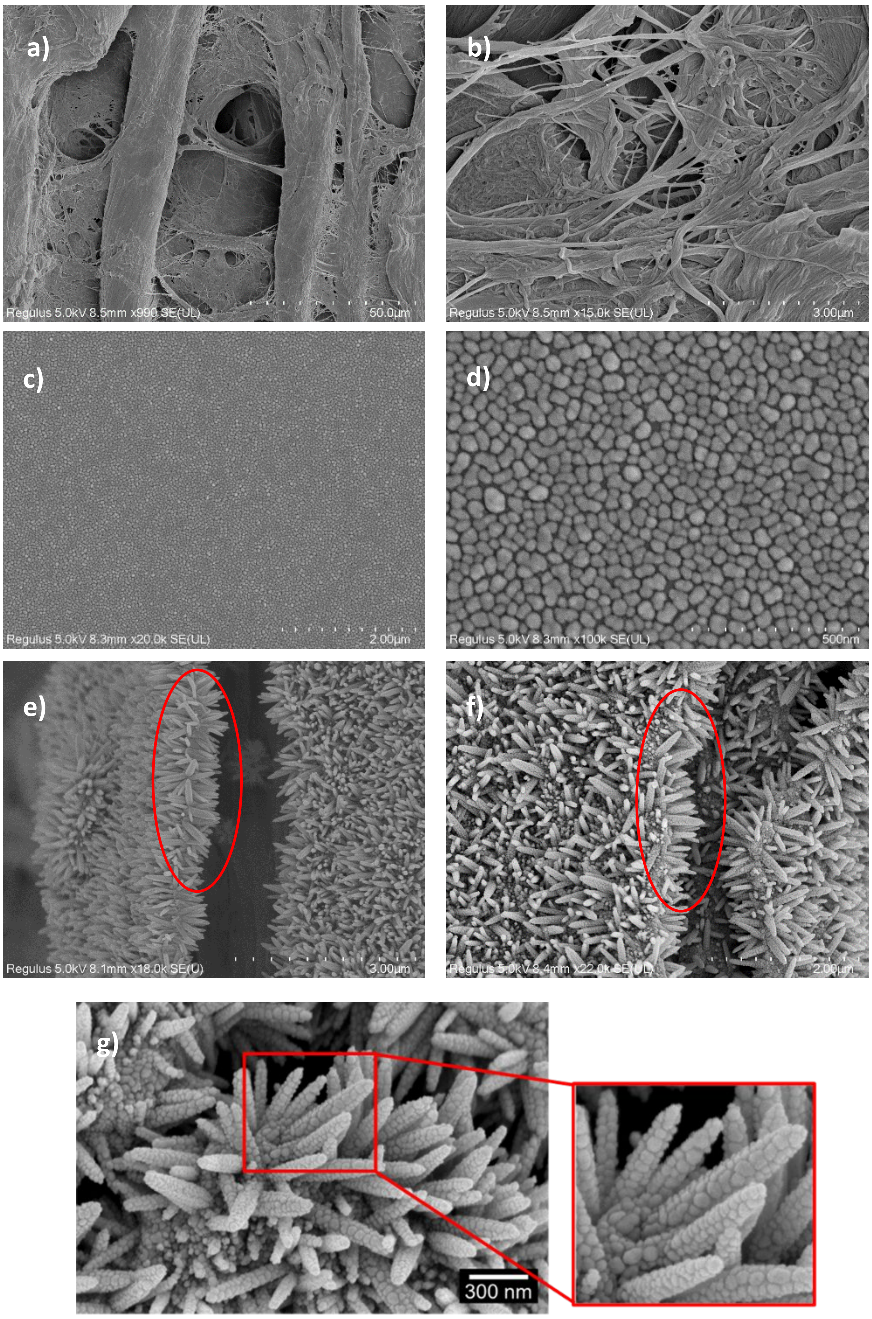

Figure S1: SEM images: a) and b) WP substrate. The WP substrates exhibit a high density of cylindrical-shaped cellulose fibers intertwined with each other. c) and d) AgNPs on silicon substrate. The diameter of the AgNPs is ~55 nm. Highlight of the profile of (e) ZnO NRs and (f) ZnO NRs/AgNPs at the edge of the paper

substrate fibers. g) Zoom of the ZnO NRs/AgNPs image highlighting the formation of distinct boundaries between the silver nanoparticles.

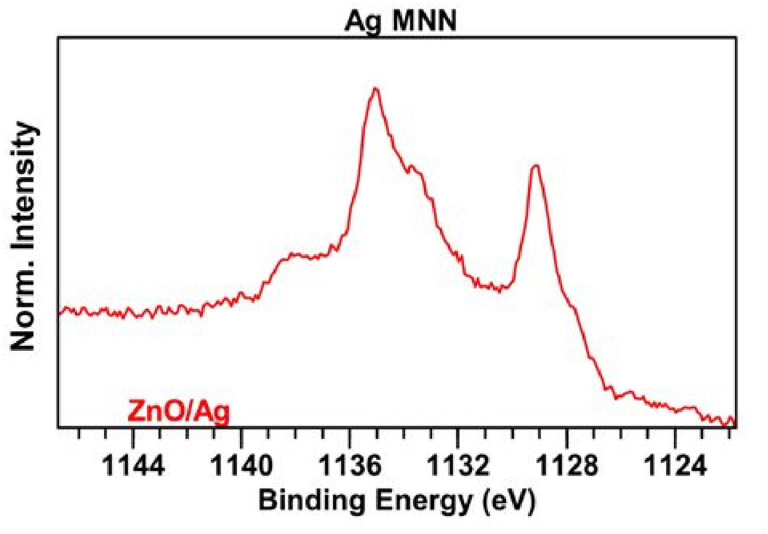

Figure S2: Ag MNN Auger emission, confirming the metallic state of silver.

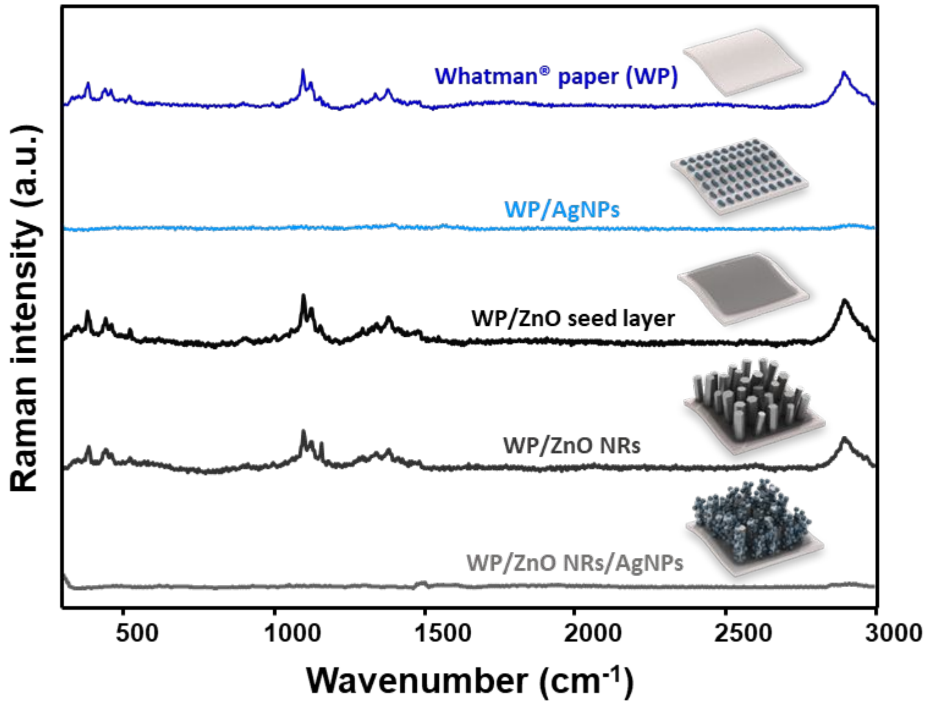

Figure S3: Raman spectra of substrates Whatman paper (WP), WP/AgNPs, WP/ZnO seed layer, WP/ZnO NRs and WP/ZnO NRs/AgNPs. The Raman spectra of all substrates show only the characteristic peaks of cellulose, and are in agreement with the literature [https://doi.org/10.1007/s10570-010-9420-z, https://doi.org/10.1021/jf304465k].

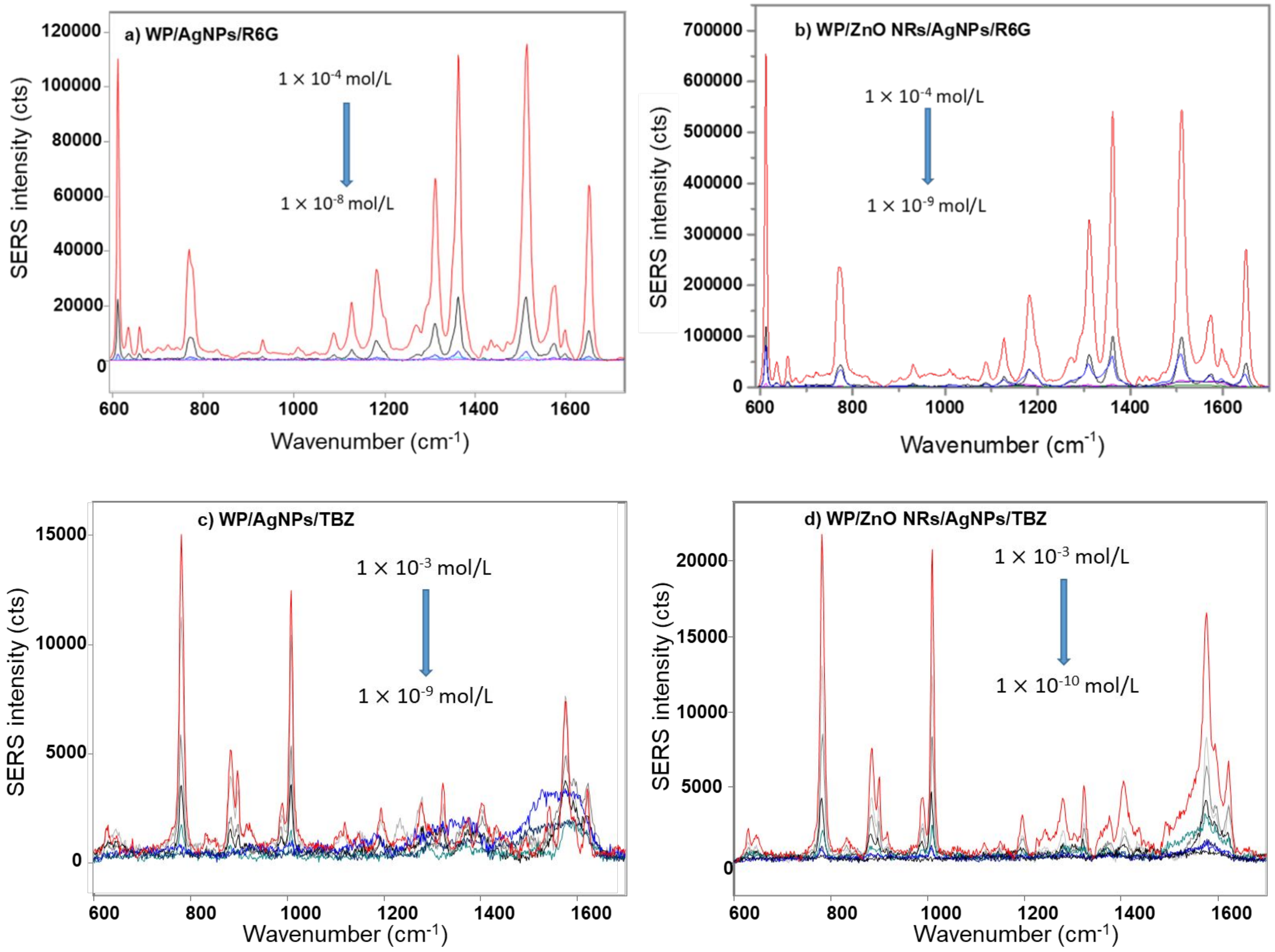

Figure S4: a)-b) SERS spectra of R6G and c)-d) TBZ on WP/AgNPs and WP/ZnO NRs/AgNPs substrates, respectively.

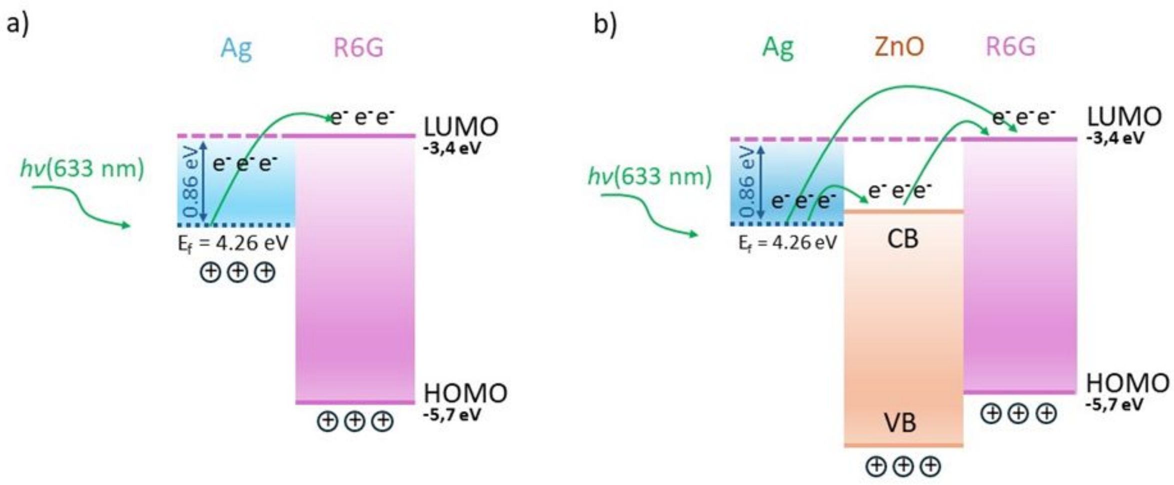

Figure S5: illustrative diagram of charge transfer process in (a) the Ag/R6G system and (b) the ZnO/Ag/R6G system.

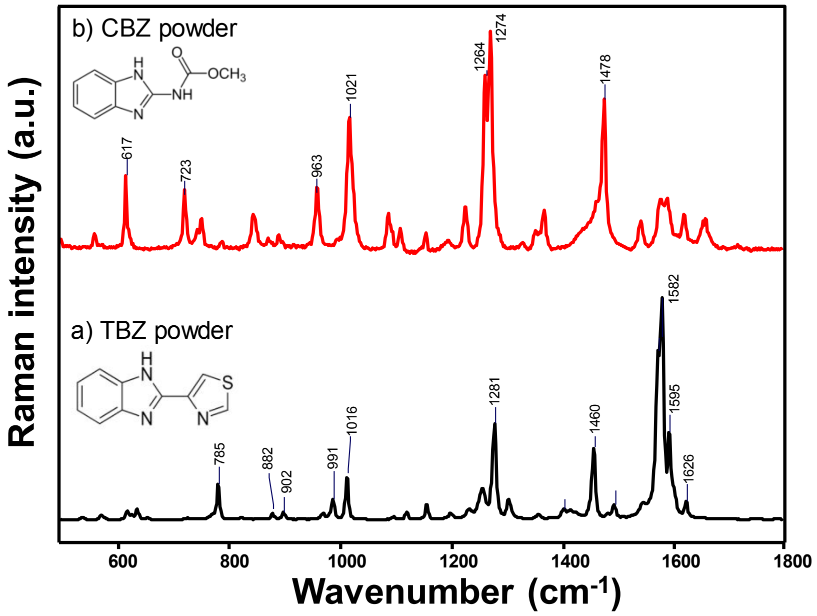

Figure S6: molecular structure and Raman spectrum of a) TBZ and b) CBZ powder. For TBZ we observed peaks at 785, 882, 902, 991, 1016, 1281, 1460, 1582, 1595, and 1626 cm^{-1}. The peaks at 785, 882, 902, and 1016 cm^{-1} are associated with the bending of C-H out-of-plane of the TBZ molecule. The peaks at 1460, 1582, 1595, and 1626 cm^{-1} result from the stretching of C=N, the peak at 991 cm^{-1} is caused by the stretching of C-S, and the stretching of the total ring is associated with the peaks at 1281 and 1582 cm^{-1} [https://doi.org/10.1016/j.talanta.2018.11.114]. For the CBZ we observe peaks at 617 cm^{-1} (ring stretching and C–C bending), 723 cm^{-1} (C–C bending and C–O–CH_3 bending), 963 cm^{-1} (C–H bending), 1021 cm^{-1} (C–N bending, C–C bending and C–O–CH_3 stretching), 1264 cm^{-1} and 1274 cm^{-1} (C–H bending and N–H bending), and 1478 cm^{-1} (C –H bending and N–H bending) [https://doi.org/10.1039/d1ra09286c].

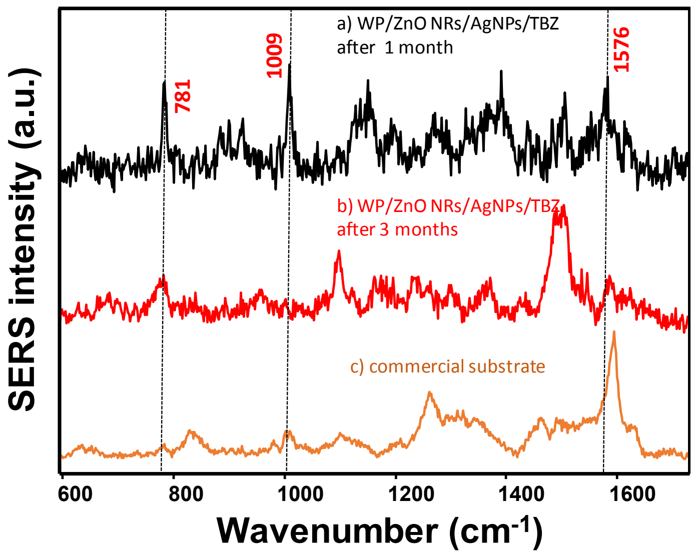

Figure S7: SERS spectra of TBZ on WP/ZnO NRs/AgNPs substrates: a) after 1 month (black spectrum) and b) after 3 months (red spectrum) following fabrication, both at a concentration of $1x10^{-7}$ mol/L. c) TBZ spectrum on a commercial Au substrate at a concentration of $1x10^{-3}$ mol/L.

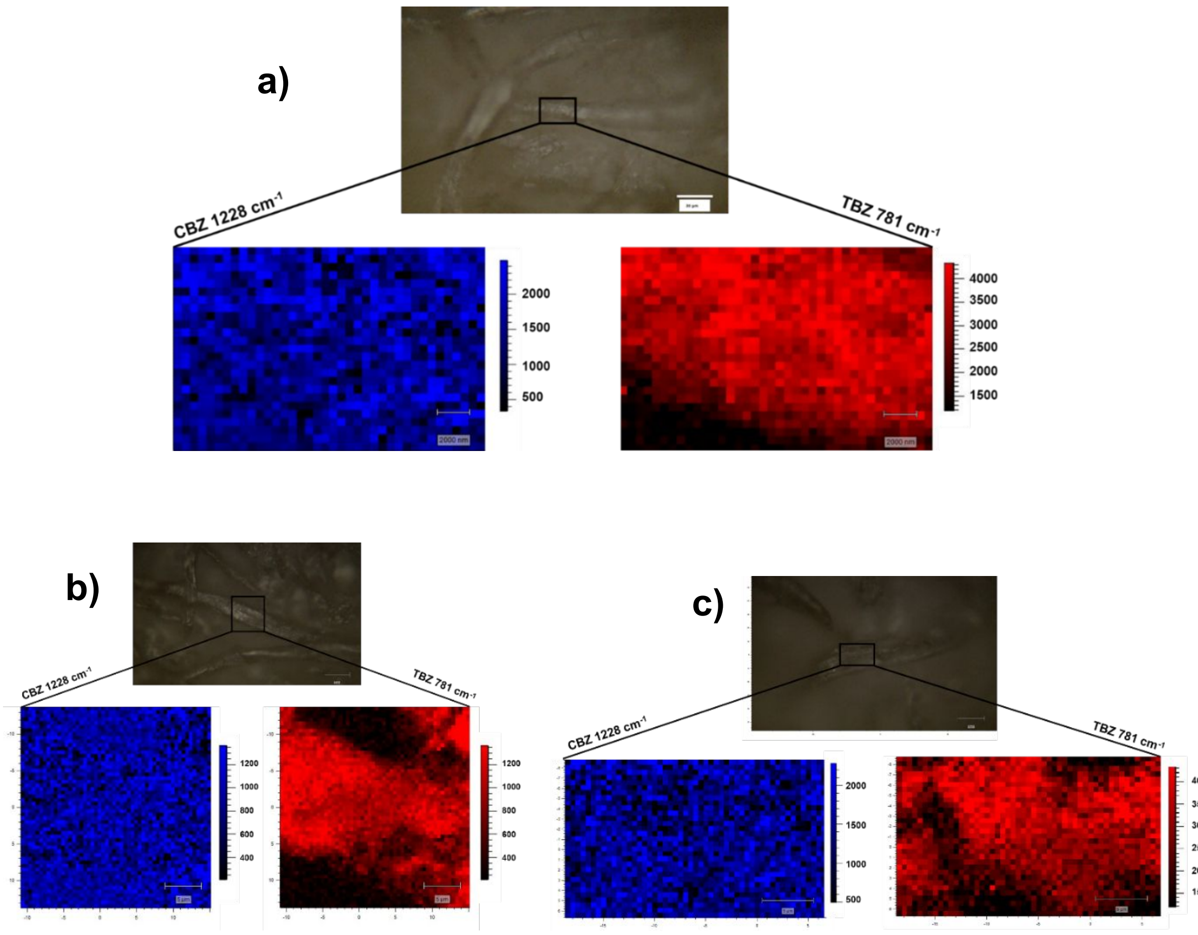

Figure S8: a) SERS mapping of the WP/ZnO NRs/AgNPs substrates containing the pesticide mixture TBZ and CBZ (TBZ + CBZ). The mapping was done over an area of 12 µm × 25 µm (total of 950 spectra). The blue spectral map corresponds to the 1228 cm⁻¹ band of CBZ. The red spectral map corresponds to the 781 cm⁻¹ band of TBZ. b) and c) SERS mapping of two different areas of the WP/ZnO NRs/AgNPs substrates containing the pesticide mixture (TBZ + CBZ). The blue spectral map corresponds to the 1228 cm⁻¹ band of CBZ. The red spectral map corresponds to the 781 cm⁻¹ band of TBZ.

Calculation of the Enhancement Factor (EF)

The enhancement factor (EF) was estimated to infer the SERS performance of the substrates as follows:

$$EF \sim \frac{\frac{I_{SERS}}{[N_{SERS}]}}{\frac{I_{RS}}{[N_{RS}]}} = \frac{I_{SERS}}{I_{RS}} \times \frac{N_{RS}}{N_{SERS}}$$

The EF in SERS quantifies the enhancement of the Raman signal intensity of molecules adsorbed onto nanoparticles (I_{SERS}) compared to the Raman signal intensity obtained in the absence of nanoparticles (I_{RS}). Thus, N_{RS} and N_{SERS} correspond to the analyte concentrations used in the conventional Raman and SERS measurements, respectively. In the case of rhodamine 6G (R6G), we used the intensity of the band at 611 cm^{-1} as a reference. For the WP/AgNPs substrate, considering the lowest concentration (1×10^{-7} mol/L) at which this band was still detectable, the average SERS intensity (I_{SERS}) was approximately 840 cts. The conventional Raman intensity (I_{RS}), measured on the paper substrate without AgNPs at a concentration of 1×10^{-4} mol/L, was around 90 cts.

Thus, the EF was calculated as:

$$EF \sim \frac{\frac{I_{SERS}}{[N_{SERS}]}}{\frac{I_{RS}}{[N_{RS}]}} = \frac{I_{SERS}}{I_{RS}} \times \frac{[N_{RS}]}{[N_{SERS}]} = \frac{840}{90} \times \frac{1x10^{-4}}{1x10^{-7}} = 9.3 \times 10^{3}$$

The same methodology was used to calculate the other enhancement factors presented in the manuscript, always considering the intensity at the lowest detectable concentration for each analyte on their respective substrates.
